# A Novel Single-Strand Specific 3′–5′ Exonuclease Found in the Hyperthermophilic Archaeon, *Pyrococcus furiosus*

**DOI:** 10.1371/journal.pone.0058497

**Published:** 2013-03-07

**Authors:** Kazuo Tori, Sonoko Ishino, Shinichi Kiyonari, Saki Tahara, Yoshizumi Ishino

**Affiliations:** Department of Bioscience and Biotechnology, Graduate School of Bioresource and Bioenvironmental Sciences, and Faculty of Agriculture, Kyushu University, Fukuoka, Japan; Keio University, Japan

## Abstract

Nucleases play important roles in all DNA transactions, including replication, repair, and recombination. Many different nucleases from bacterial and eukaryotic organisms have been identified and functionally characterized. However, our knowledge about the nucleases from Archaea, the third domain of life, is still limited. We searched for 3′–5′ exonuclease activity in the hyperthermophilic archaeon, *Pyrococcus furiosus,* and identified a protein with the target activity. The purified protein, encoded by PF2046, is composed of 229 amino acids with a molecular weight of 25,596, and displayed single-strand specific 3′–5′ exonuclease activity. The protein, designated as PfuExo I, forms a stable trimeric complex in solution and excises the DNA at every two nucleotides from the 3′ to 5′ direction. The amino acid sequence of this protein is conserved only in *Thermococci,* one of the hyperthermophilic classes in the Euryarchaeota subdomain in Archaea. The newly discovered exonuclease lacks similarity to any other proteins with known function, including hitherto reported 3′–5′ exonucleases. This novel nuclease may be involved in a DNA repair pathway conserved in the living organisms as a specific member for some hyperthermophilic archaea.

## Introduction

The stability of DNA largely depends on the accuracy of its repair systems, which remove DNA damage induced by exogenous and endogenous agents or introduced by DNA metabolism, such as replication [1], [2]. If unrepaired or misrepaired, DNA damage can cause cell death. To mend the many different types of DNA damage, cells utilize DNA repair systems, including homologous recombination repair (HR), mismatch repair (MMR), and excision repair (NER, BER) [3], [4].

Archaea, the third domain of life, has DNA replication factors that resemble those in Eukarya with respect to the amino acid sequences, suggesting that the mechanisms of DNA replication would be similar in eukaryotic and archaeal cells [5]–[7]. The DNA repair proteins are also conserved between Archaea and Eukarya, and structural studies of the archaeal homologs have yielded numerous important insights into the structures and functions of eukaryotic DNA repair proteins [8]–[12]. In addition, the DNA repair systems in the hyperthermophilic archaea are especially attractive to investigate, for understanding the phenomena allowing life to exist at extremely high temperatures. It has been suggested that thermophiles must have extremely efficient and specialized DNA repair systems to withstand not only the high temperatures but also other damaging factors, such as ionizing and ultraviolet radiation and chemical agents. The spontaneous mutation rate is accelerated at elevated temperatures, due to the formation of lesions in the DNA bases [13]–[15].

*Pyrococcus furiosus* is a hyperthermophilic archaeon that optimally grows at over 100°C under anaerobic conditions, and its DNA transactions have been partially characterized [9], [16], [17]. The exposure of *P. furiosus* cells to ionizing radiation reportedly resulted in chromosomal fragmentation, but a following incubation of the cells at 95°C resulted in chromosome reassembly [14]. These findings suggested that *P. furiosus* must have a highly efficient DNA repair system for DNA strand breaks, but the details about the components and mechanism of the system still remain unknown.

Deoxyribonucleases (DNases) operate as central components of most DNA repair systems, by executing or initiating DNA damage removal to promote cell survival and ensure genetic integrity [18], [19]. DNases can be classified into structure-, damage-, or sequence-specific families, with respect to their substrate preferences. They can also be divided into exonucleases, which hydrolyze nucleic acids from either the 5′ or 3′ end, and endonucleases, which hydrolyze internal phosphodiester bonds without the requirement of a free DNA end. Many exonucleases have been identified in Eukarya and Bacteria [18], and their functions have been analyzed both *in vitro* and *in vivo*. Single-stranded DNA specific exonucleases are particularly abundant in *E. coli*, where they play important roles in several repair processes within the cell, such as MMR [4], HR [20], and tandem repeat stabilization [21]. Although similar functional exonucleases are expected to be involved in the repair systems in *P. furiosus*, no single-stranded DNA specific 3′–5′ exonuclease has been identified yet, except for the DNA polymerase-associated exonuclease activity [22], [23]. To understand the DNA repair systems in more detail in the hyperthermophilic archaea, the unknown factors, including DNases, must be identified.

In this study, we identified a novel nuclease in *P. furiosus* cells. This enzyme degrades single-stranded DNA, but not double-stranded DNA, from the 3′ to 5′ direction. No conserved sequence motifs for the 3′–5′ exonucleases were detected in the deduced amino acid sequence of this protein. Furthermore, genes encoding the homologous sequence of this enzyme are present only in the *Thermococci* in Archaea annotated as hypothetical protein in the public databases, and therefore, it is a novel nuclease.

## Results

### Identification of a Nuclease Activity in the Heat-stable Protein Library from *P. furiosus*

**Figure 1:**
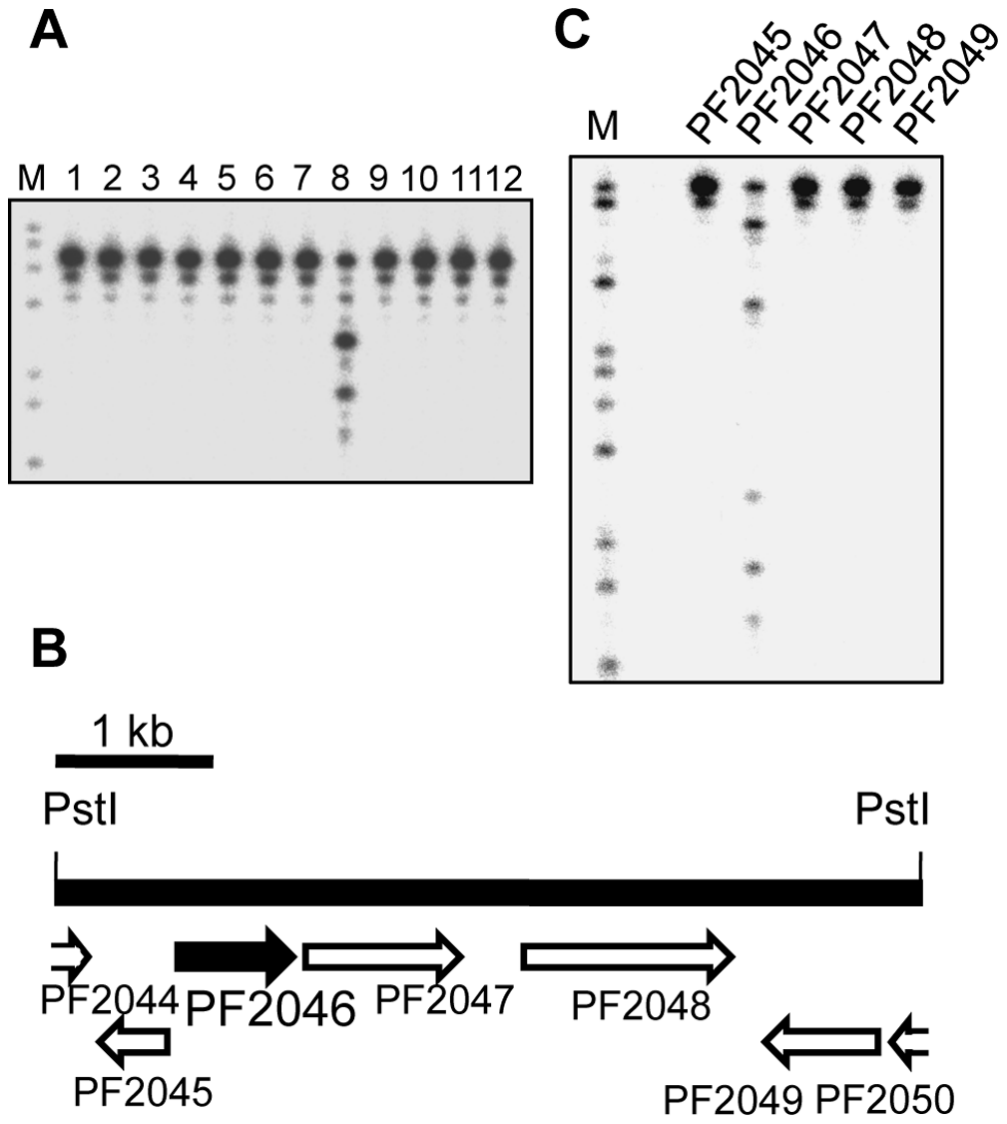
Screening and identification of the gene responsible for the DNA cleavage activity. (A) The *E. coli* cells, transformed with pUC118 containing each PstI-digested DNA fragment from the positive cosmid clone, were heated. The heat-stable cells extracts from each clone were assayed for DNase activity, using ^32^P-labeled d27 as the substrate. The reactions were analyzed by PAGE on a 12% gel containing 8 M urea followed by autoradiography. Lane M shows the GA ladder of d49R, generated by the Maxam-Gilbert reaction. The other lanes (1–12) are labeled d27 treated with heat-stable cell extracts from different clones. (B) The ORFs identified on the PstI-DNA fragment obtained from the positive clone #8. (C) The 5 full-length ORFs on the *Pst*I fragment in the positive clone were individually cloned and expressed in *E. coli*, and the same DNase assay was performed using ^32^P-labeled d49R as the substrate. PF2046 was identified as the gene responsible for the DNA cleavage activity.

We searched the databases for an ORF bearing a sequence homologous to those of the known 3′–5′ exonucleases in the *P. furiosus* genome, to detect homologs of known exonucleases. However, no homologous sequence was found. Therefore, we tried to identify a novel enzyme containing a 3′–5′ exonuclease by screening for the activity from the heat-stable protein libraries derived from *P. furiosus,* as described in the Materials and Methods section [24]. Among 500 independent heat extracts of *E. coli* clones transformed by the cosmid-based gene library of *P. furiosus*, we isolated a clone producing a protein that degrades the synthetic oligonucleotide. The cosmid DNA containing a *P. furiosus* genomic DNA of 35 kbp was recovered from the *E. coli* clone exhibiting the activity. The inserted genomic DNA fragment was subcloned into the pUC118 plasmid vector after PstI digestion. Then, as a second screening, we searched for the nuclease activity in the heat-resistant cell extract from each transformant. One clone with the target activity was found (lane 8 in Fig. 1A). The plasmid was prepared from clone #8, and the inserted fragment (about 5 kbp) was sequenced. As shown in Fig. 1B, 7 open reading frames (ORFs), among which 5 were full-length and 2 were truncated, were identified. Therefore, we cloned these 5 full-length ORFs individually into the pET21a expression vector, to determine which ORF is responsible for the degradation of ssDNA. As shown in Fig. 1C, the heat extract of the *E. coli* transformant containing PF2046 clearly expressed the nuclease activity.

### Purification of the Recombinant Protein Encoded by PF2046

**Figure 2:**
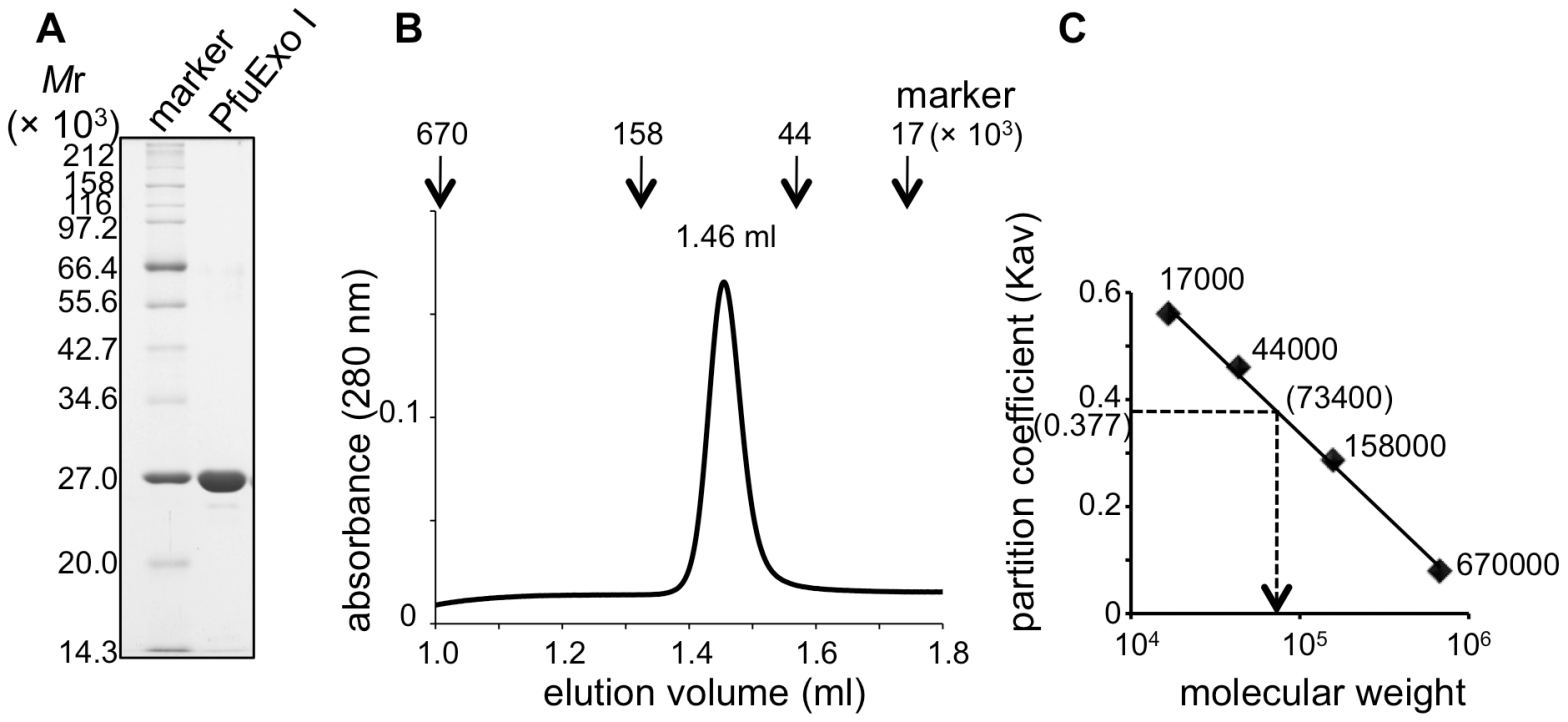
Purification and oligomeric state of PfuExo I. (A) The purified PfuExo I protein (2 µg) was subjected to 12% SDS-PAGE, and the gel was stained by Coomassie Brilliant Blue. Marker, molecular mass standards (New England Biolabs Inc.). (B) The oligomeric states of PfuExo I were analyzed by gel filtration. The elution profiles, monitored by the absorbance at 280 nm, are shown. The peak positions of the marker proteins from a parallel chromatographic analysis are indicated by the arrows at the top of the chromatogram. (C) The standard curve was obtained with marker proteins. The molecular weight of each marker protein is shown with the plots. The molecular weight of PfuExo I, calculated from its *K*av value, is shown.

The recombinant protein encoded by PF2046 was successfully overproduced, by cultivating the *E. coli* cells bearing the expression plasmid, pPF2046, as described above, with IPTG induction. The protein was purified to near homogeneity (Fig. 2A) by the three sequential chromatography steps described in the Materials and Methods section. From a one liter culture (3.4 g cells), 1.5 mg of homogeneous protein was obtained. The relative molecular mass of the purified protein determined from its migration position in the SDS-PAGE gel corresponded to 25,596, calculated from the deduced amino acid sequence. We designated the protein as PfuExo I.

### The Oligomeric State of PfuExo I

To investigate the oligomeric state of PfuExo I in solution, analytical gel filtration chromatography was performed (Fig. 2B). The molecular weight of PfuExo I was estimated from the elution profile to be approximately 73,400 (Fig. 2C). As the theoretical molecular mass of PfuExo I is 25,596, as described above, the gel filtration results suggested that PfuExo I exists in a homotrimeric form in solution.

### Detection of PfuExo I in the *P. furiosus* Cells

**Figure 3:**
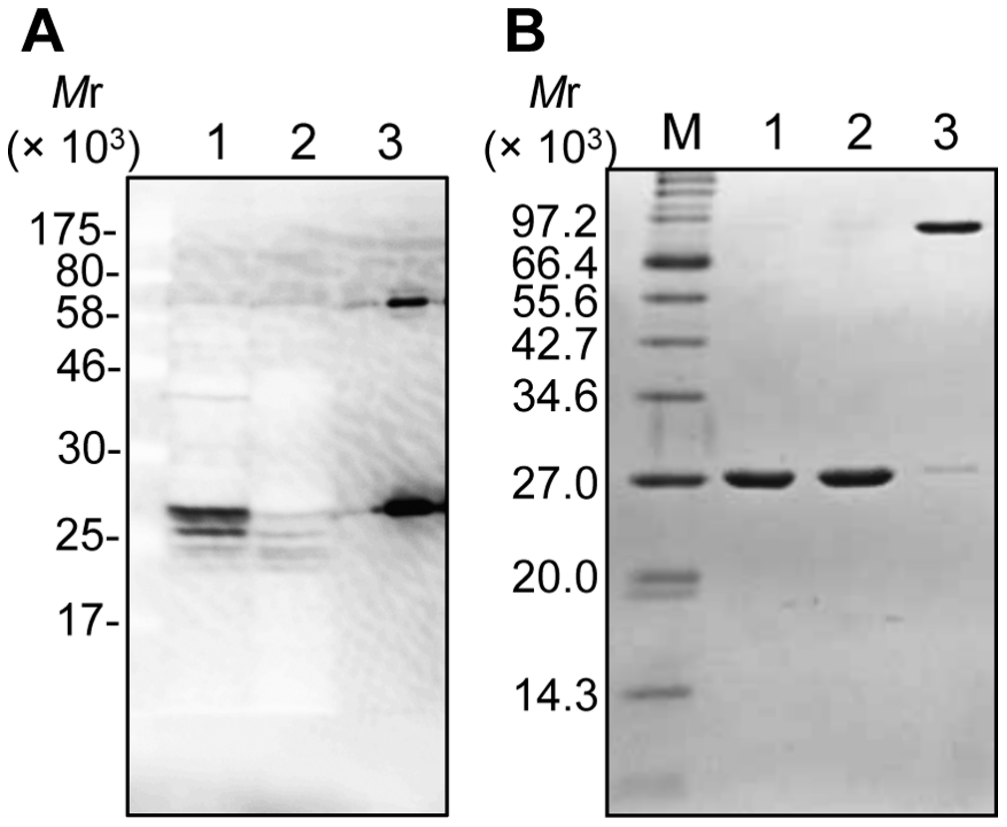
Detection of PfuExo I in *P. furiosus* cells. (A) *P. furiosus* cells in the exponential growth phase (lane 1) and the stationary phase (lane 2) were harvested, and the whole cell extracts from 6×10^8^ cells, as well as 10 ng of the recombinant protein (lane 3), were subjected to 14% SDS-PAGE, followed by western blot analyses using anti-PfuExo I antiserum. (B) The recombinant proteins were boiled in the loading solution, containing 50 mM Tris-HCl, pH 6.8, 1% SDS, 1% 2-mercaptoethanol, and 10% glycerin, for 10 min (lane 1), 5 min (lane 2), and 0 min (lane 3), respectively, and subjected to 14% SDS-PAGE. The gel was stained by Coomassie Brilliant Blue.

Using the highly purified PfuExo I protein, a polyclonal antibody was prepared. A western blotting analysis revealed that a protein band corresponding to the size of the recombinant PfuExo I (25,596), which specifically reacted with the antibody, was detected in the cell extract prepared from an exponential phase *P. furiosus* cell culture (Fig. 3A). This band was not detected in the cell extract prepared from the stationary phase culture of the cells, probably because of physiological proteolysis. The smaller bands, in addition to the main band, detected in the exponential phase may indicate that the function of PfuExo I is regulated with relatively short turnover in the *P. furiosus* cells. The appearance of an immunoreactive band at around 80,000 (Fig. 3A) is consistent with the results from the SDS-PAGE of the recombinant PfuExo I without heat treatment (Fig. 3B). The protein at 80,000 is also likely to be PfuExo I that was not completely denatured. The trimeric structure of this protein should be extremely stable, even in the presence of SDS.

### Properties of the Exonuclease Activity of PfuExo I

**Figure 4:**
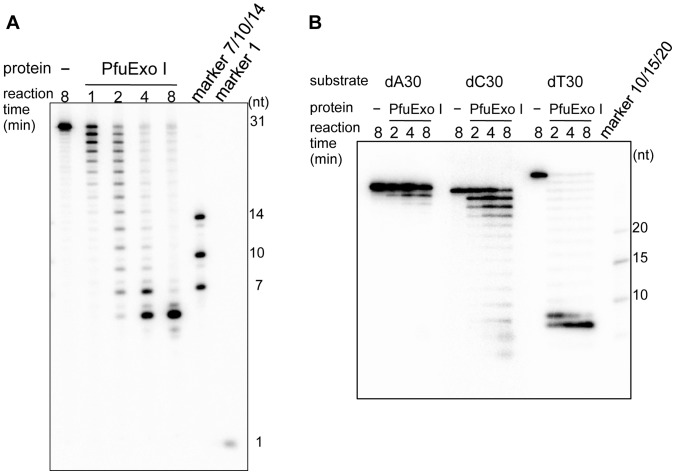
DNA cleavage activity of PfuExo I. (A) Time course experiments of the DNase activity of PfuExo I were performed, using dAC31 ssDNA labeled with ^32^P at the 5′-end. PfuExo I (10 nM, as a trimer) was incubated with 5 nM DNA at 65°C. For each time point, aliquots were removed from the reaction and quenched. These samples were subjected to PAGE on an 18% gel containing 8M urea, and the degradation products were visualized by autoradiography using TyphoonTrio (GE Healthcare). The 7-nt, 10-nt, and 14-nt oligonucleotides with the same sequence as dAC31, and [γ-^32^P]ATP were loaded alongside to provide markers. (B) dA30, dC30, and dT30 were used as substrates with 5 nM of PfuExo I. The samples were subjected to PAGE on a 12% gel containing 8M urea, and the degradation products were visualized by autoradiography. The 10-nt, 15-nt, and 20-nt poly dAs were loaded alongside to provide markers.

The nuclease assay was performed using highly purified PfuExo I and a 31 nucleotide (nt)-long 5′-^32^P-labeled deoxyoligonucleotide. As shown in Fig. 4A, the deoxyoligonucleotide substrate became shorter as the reaction time progressed. This result clearly supports the proposal that the purified PF2046 protein actually has 3′-exonuclease activity. Similar to other nucleases, the activity of PfuExo I was dependent upon a divalent cation, and Mg^2+^ was the most preferable (data not shown). Ladder-like bands were detected on the gel, indicating the progress of the cleavage reaction. It is interesting that the ladder bands corresponded to exactly every two nucleotides judged from the positions of the size marker bands, 7, 10, and 14 mers with the sequence same as that of substrate DNA. Since the DNA substrate with the (AC)-repeated sequence was used for the nuclease assay, some nucleotide preference may exist for the cleavage by PfuExo I. Therefore, homooligomers were used as the substrates for the PfuExo I reaction. As shown in Fig. 4B, the homo dT 30 nt-long was the most preferable substrate for PfuExo I. The dC 30 nt-long DNA was the second best substrate, and the dA 30 nt-long was the least preferred. The dG 30 nt-long oligonucleotide could not be tested, because it could not be appropriately synthesized. These results support the idea that the ladder bands in Fig. 4A result from cleavage at the position of dC. However, the ladder bands in Fig. 4B also correspond to every two nucleotides. One more notable property of PfuExo I is that the degradation of the DNA strand does not proceed to the 5′-end, but stops at the 4^th^–6^th^ nucleotide from the 5′-end. This probably occurs because PfuExo I needs to grasp a 4–6 nt-long DNA strand to express its nucleolytic activity.

**Figure 5:**
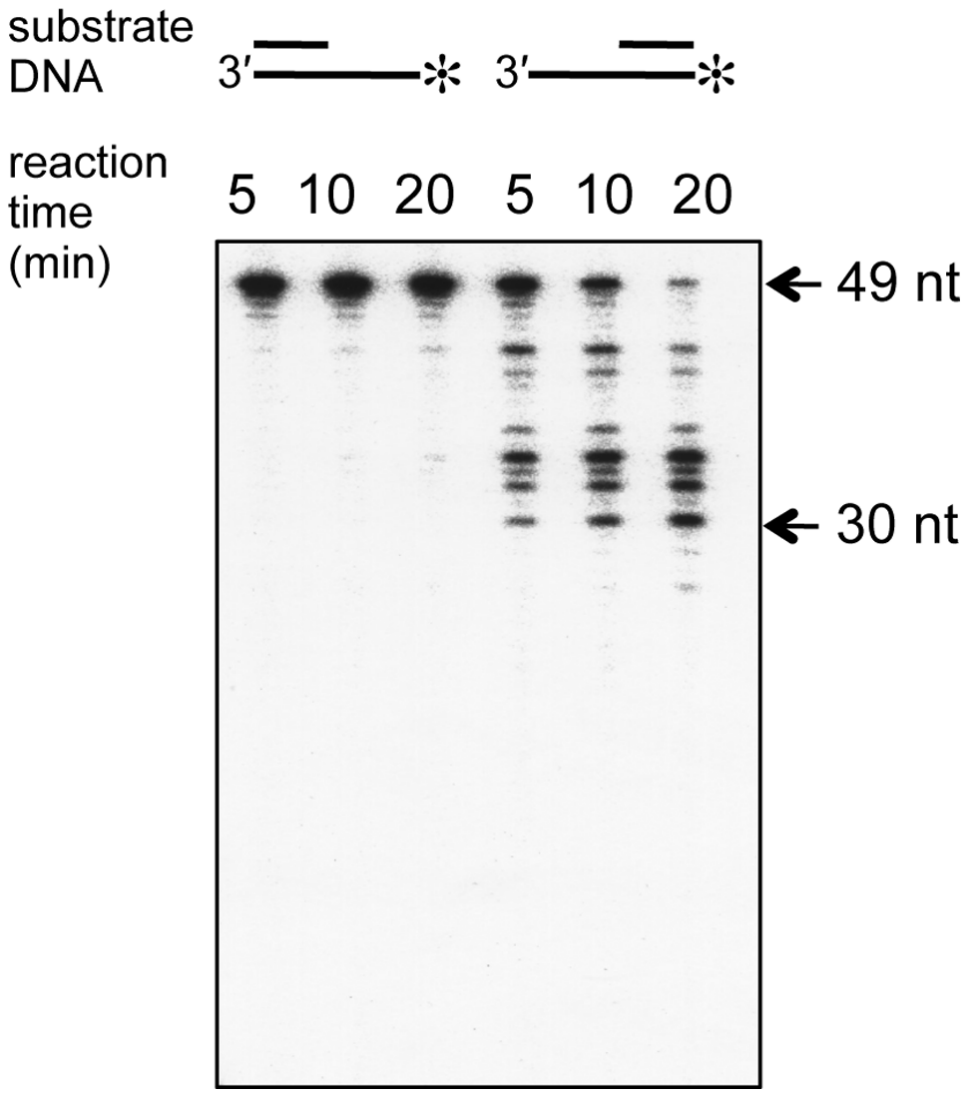
Cleavage specificity of PfuExo I. Purified PfuExo I (10 nM) was incubated with 5′- overhang and 3′-overhang DNAs (5 nM) for various times at 55°C. The positions labeled by ^32^P are indicated by an asterisk for each substrate. Aliquots were removed from the reactions, quenched, and then resolved by PAGE on a 12% gel containing 8 M urea.

To obtain further evidence that PfuExo I degrades the single-stranded DNA in the 3′ to 5′ direction, the nuclease assays were performed using 3′ and 5′- overhang DNAs. As shown in Fig. 5, the reaction products were detected only from the 3′- overhang DNA substrate, and the cleavage reaction stopped at the 30-mer. This is the position of the 3^rd^ nucleotide from the end of the double-stranded region. This result supports the proposal that PfuExo I is an exonuclease with 3′ to 5′ polarity, and its substrate DNA must be at least a few nucleotides long for PfuExo I to bind.

It is interesting to analyze the degradation mode of the PfuExo I from the enzymological aspect. We did several experiments investigating how processive this enzyme is for its cleavage of the DNA strand. Constant PfuExo I and increasing amounts of substrate DNA were reacted. The cleavage pattern showed that the amounts of DNA strands with shorter sizes decreased with the increasing amounts of substrate DNA (data not shown). This result and other experiments suggested that the cleavage mode of PfuExo I is distributive.

### Examination of the Endonuclease Activity of PfuExo I

**Figure 6:**
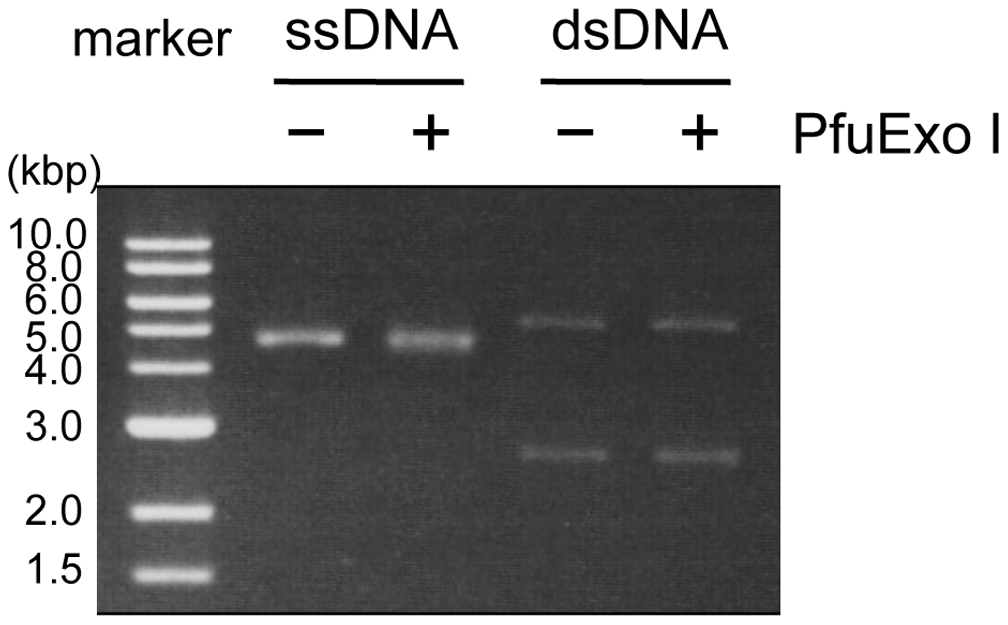
Endonuclease activity of PfuExo I. Purified PfuExo I was incubated with circular ssDNA (M13 mp18) or dsDNA (pBR322) in the reaction condition described in Materials and Methods. Reaction products were analyzed by electrophoresis on a 1% agarose gel, followed by ethidium bromide staining.

To determine whether PfuExo I also has endonuclease activity, endonuclease assays were performed using the circular forms of single- and double-stranded DNAs. As shown in Fig. 6, no cleavage was detected with either ssDNA or dsDNA, even after a 30-min incubation. This result indicated that PfuExo I requires the terminus to express the nuclease activity.

### Comparison of the Binding Affinities of PfuExo I to Various DNA Substrates

**Figure 7:**
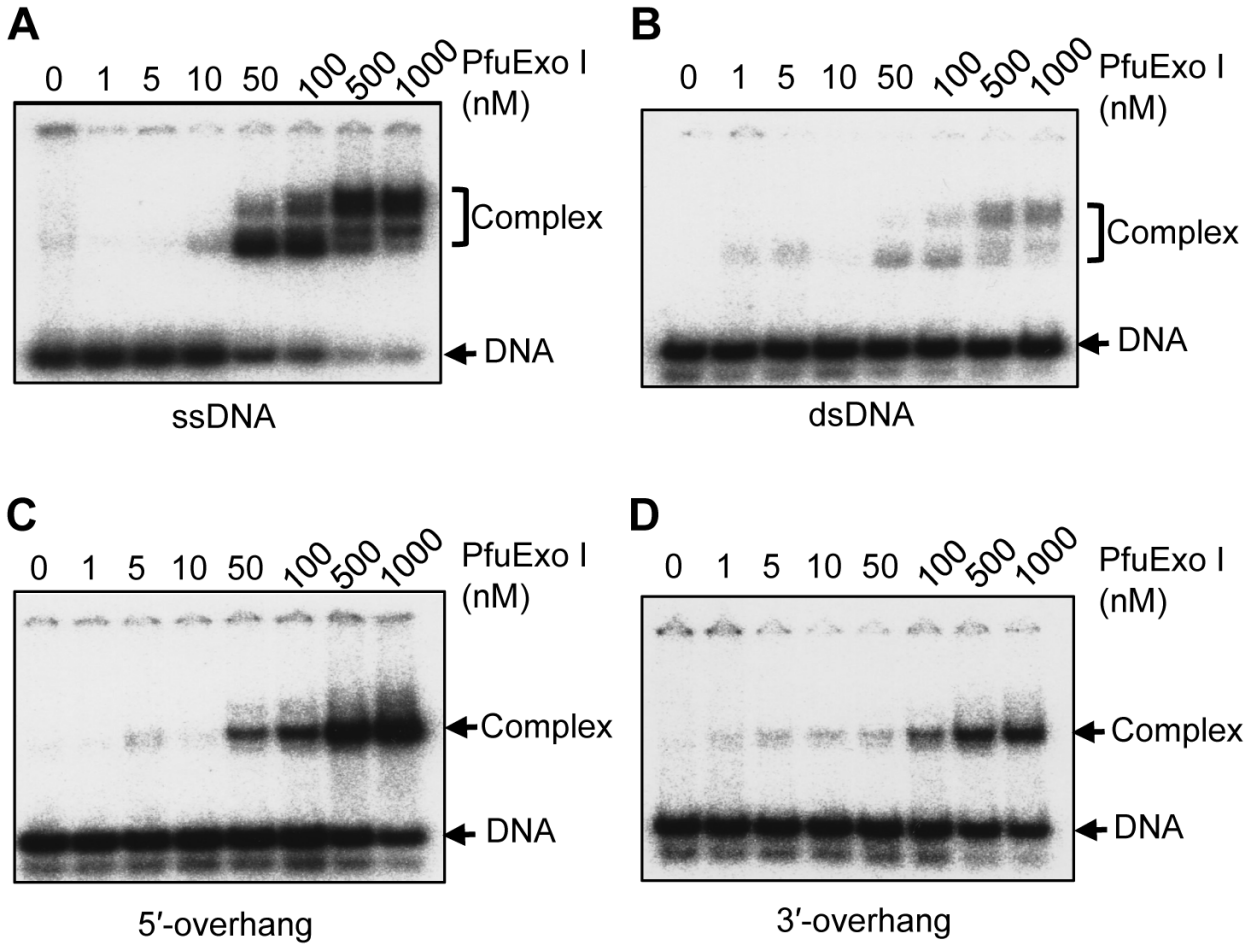
DNA binding activity of PfuExo I. Various concentrations (1, 5, 10, 50, 100, 500, or 1000 nM) of PfuExo I were incubated with ^32^P-labeled ssDNA (A), dsDNA (B), 5′-overhang DNA (C), or 3′-overhang DNA (D). The protein-DNA complexes were separated by 4.5% PAGE and visualized by autoradiography.

To further characterize PfuExo I, gel mobility shift assays were performed using different types of DNA in the absence of Mg^2+^, to prevent substrate loss due to degradation, as shown in Fig. 7. The shifted bands should correspond to the DNA-PfuExo I complexes. Multiple shifted bands were observed with the ssDNA substrate (Fig. 7A). As slower migrating complexes were observed with increasing protein concentrations, PfuExo I may bind ssDNA nonspecifically at higher concentrations. Slight shifts were observed when dsDNA was used (Fig. 7B). Shifted bands were distinctly observed from both the 3′- and 5′-overhang DNAs, but only one band was observed (Fig. 7C, and 7D), suggesting that the preferential binding of PfuExo I occurred at the single-stranded region, which is not long enough for the second binding. These observations are consistent with the results of the nuclease assays, showing the single-stranded specific cleavage. Various lengths of ssDNAs were used for the gel shift assays and distinct shift band was observed from the ssDNA of 7 nt-long (data not shown), supporting that 7 nt-long is sufficient for PfuExo I to stably grasp DNA.

### PfuExo I Homologs in Archaea

**Figure 8:**
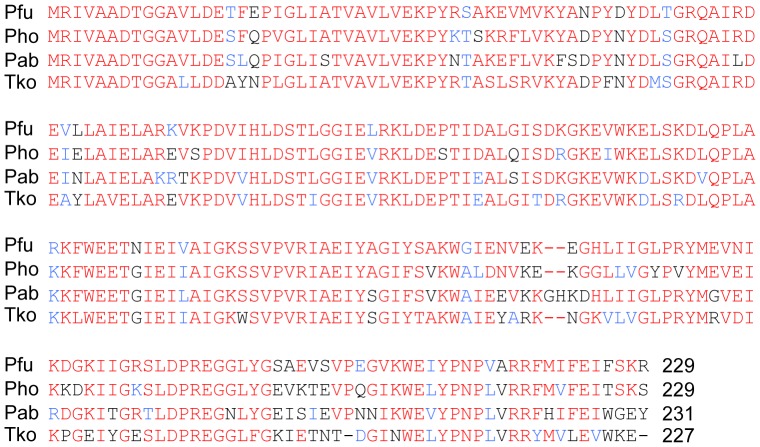
Comparison of the PfuExo I amino acid sequence with those of homologs found in Thermococcaceae. Pfu, *P. furiosus*; Pho, *P. horikoshii*; Pab, *P. abyssi*; Tko, *T. kodakarensis*. Identical and similar amino acid residues are indicated by red and blue letters, respectively.

The deduced amino acid sequence of PfuExo I is not similar to any proteins from eukaryotic and bacterial organisms in the public databases. From a comprehensive search of the public databases, ORFs with highly similar sequences were detected only from the organisms that belong to the *Thermococcaceae* (6 *Pyrococcus* and 10 *Thermococcus* species) in the Archaeal domain. The multiple alignment of the ORF sequences from *P. furiosus*, *P. horikoshii*, *P. abyssi*, and *T. kodakarensis* is shown in Fig. 8. The sequence identities among these proteins are 70% on average. The newly discovered nuclease likely functions in DNA repair in Thermococcales lineage. It would be interesting if these organisms have a specific DNA repair system including this protein for the DNA damages occurring due to the high temperatures of their habitat.

## Discussion

Archaea are unique organisms that are evolutionally different from bacterial and eukaryotic organisms. To understand their DNA repair systems, several archaeal proteins homologous to the eukaryotic and bacterial proteins involved in the DNA repair processes have been identified and biochemically analyzed to date. The hyperthermophilic archaea should have especially efficient DNA repair systems, due to their habitation in extreme environments. However, the DNA repair system in Archaea is still not well understood, and many more efforts are required to determine the similarities and differences between the archaeal DNA repair system and those of Bacteria and Eukarya. For example, the thermophilic archaea lack the NER damage-recognition proteins (eukaryotic XPA and XPC or bacterial UvrA and UvrB) and the MMR-specific proteins (MutS and MutL) [25]. Every factor that seems to be involved in DNA repair systems must be identified to elucidate the molecular mechanisms of damaged DNA repair in Archaea, and the exonucleases definitely play important roles in the processes. It would be especially interesting to determine whether any unique repair ability is present in the third domain of life.

In *E. coli* cells, several proteins function as single-stranded DNA specific exonucleases. RecJ, Exo I, Exo VII, and Exo X are involved in MMR and HR. In Archaea, a 5′-3′ exonuclease, NurA, is conserved only in the thermophilic archaea, and the *nurA* gene is organized in an operon structure with *rad50* and *mre11*, which are involved in double-stranded break repair [26]. This nuclease probably plays an important role in generating 3′ single-stranded DNA during archaeal HR, together with Mre11 and Rad50. HerA, a bipolar DNA helicase, is also present in the operon, and is involved in this DNA processing system [27]. In addition, several genes with sequences similar to that of the bacterial RecJ nuclease are present in the archaeal genomes [28]. A recent report showed that one of the RecJ homologs in *T. kodakarensis* stably interacts with the GINS complex, an essential factor for both the initiation and elongation processes in DNA replication, and its 5′-3′ exonuclease activity is stimulated by the interaction with GINS [29]. The authors designated this protein as GAN (GINS-associated nuclease), and proposed that GAN is involved in lagging strand processing. It is still not known if the bacterial RecJ-like proteins are involved in some repair system in the archaeal cells.

This is the first report to describe a single-stranded specific 3′–5′ exonuclease in Archaea. The amino acid sequence of the identified protein lacks obvious similarity to the known 3′–5′ exonucleases, which have some conserved motifs [18], and therefore, it is a new nuclease family member. At this point, it is not easy to predict the exact function of this nuclease, since it has no homolog in either Bacteria or Eukarya. The genes encoding sequences homologous to this enzyme are found only in the *Thermococcales,* although more than 140 archaeal genomes have been completely sequenced. It is most likely that the DNA repair systems are conserved in the living organisms, but the diverse members are involved in these processes in various organisms. However, because of the specific habitation, the organisms in *Thermococcales* may have a unique pathway for nucleic acid metabolism. The DNA of hyperthermophilic archaea is known to be extremely resistant to breakage *in vivo* by radiolysis and thermolysis. DiRuggiero *et al*. reported that the amount of mRNA for PF2046, corresponding to PfuExo I, increased after ionizing irradiation [30]. The fact that the chromosomal fragmentation occurring upon the exposure of *P. furiosus* cells to ionizing radiation was quickly ameliorated by an incubation of the cells at 95°C [14] suggests that *P. furiosus* must have a highly efficient DNA repair system for DNA strand breaks. PfuExo I may be one of the crucial enzymes in this pathway. Ionizing radiation, radiomimetic drugs, and to some extent, all free radical-based genotoxins induce DNA double-strand breaks by oxidative fragmentation of DNA sugars. Most of the breaks bear terminal 3′-phosphate or 3′-phosphoglycolate moieties [31]–[33]. Although we examined the end-processing activity of PfuExo I using synthetic oligonucleotides with a phosphate at the 3′-end, the enzyme could not excise ssDNA (data not shown). Therefore, another unknown factor, such as a phosphatase, may be required to remove the 3′ phosphate before PfuExo I functions, if this nuclease participates in end-processing.

To prove that PfuExo I is actually involved in some DNA repair system in *P. furiosus,* genetic studies using a mutant strain lacking or deficient in the encoding gene are necessary. A few genetic investigations have been performed on the DNA repair genes in Archaea. RadA functions in the repair of DNA damaged by UV and methylmethane sulfonate [34], and Mre11 is important for the repair of DNA double-strand breaks, whereas Rad50 is dispensable [35] in Haloarchaea. In addition, the *uvrA*, *uvrB* and *uvrC* genes are required for the repair of ultraviolet light-induced DNA photoproducts in Haloarchaea [36]. In hyperthermophilic Archaea, gene disruption methods and genetic tools were first developed for the crenarchaea of the genus *Sulfolobus* [37]. These genetic tools were used to analyze the functions of the genes involved in the DNA replication and repair processes in *S. islandicus* [38]. For anaerobic hyperthermophilic euryarchaea, genetic tools for gene targeting have been established in *T. kodakarensis* [39], [40]. We obtained knock-out mutants of the genes encoding the proteins Xpb, Xpd, Hef, Hjc, and Hjm, and investigated the sensitivity of all of these mutants to several different types of DNA damaging agents, including UV, γ-rays, mitomycin C, and methylmethane sulfonate [41]. In the same study, we also proposed that the *radA, mre11, rad50, herA, and nurA* genes are essential, because deletion strains for these genes could not be obtained. The HR event may be especially important for repairing the DNA damage caused by the high temperature required for *T. kodakarensis* viability. Using this technique, we could make a deletion mutant of the homolog of PfuExo I in *T. kodakarensis* (TK1646) and characterize it. This experiment is now underway. *P. furiosus* cells with high transformation efficiency have been described recently [42], [43], and therefore, the creation of PF2046-disrupted cells is also possible.

## Materials and Methods

### DNA Substrates

The following oligonucleotides were synthesized by Sigma-Aldrich Japan K. K. (Tokyo, Japan): d22, 5′-AATTCGTGCAGGCATGGTAGCT-3′; d27, 5′-AGCTATGACCATGATTACGAATTGCTT-3′; d49F, 5′-AGCTACCATGCCTGCACGAATTAAGCAATTCGTAATCATGGTCATAGCT-3′; d49R, 5′-AGCTATGACCATGATTACGAATTGCTTAATTCGTGCAGGCATGGTAGCT-3′; dAC31, 5′-ACACACACACACACACACACACACACACACA-3′; dA30, 30-nt poly dA; dT30, 30-nt poly dT; and dC30, 30-nt poly dC. These oligonucleotides were labeled at the 5′ termini with [γ-^32^P]ATP by T4 polynucleotide kinase (New England Biolabs). The 5′- overhang DNA (d27 and d49F), 3′- overhang DNA (d22 and d49F), and dsDNA (d49F and d49R) were prepared by annealing the oligonucleotides in TAM buffer, containing 40 mM Tris-acetate, pH 7.8, and 0.5 mM magnesium acetate.

### Cloning and Identification of the PfuExo I Gene

The cosmid-based genomic library, in which each of clone contains the *P. furiosus* DNA fragment with 35–40 kbp, was prepared as described previously [24]. Then, heat-stable protein extracts were obtained from 500 independent clones by the heat treatment at 80°C for 10 min. The heat-stable extracts were used for the screening of DNase activity. Cosmid DNA was prepared from the clone exhibiting the heat-stable DNase activity, and was partially digested by PstI. The DNA fragments were inserted into pUC118, and the resultant plasmids were introduced into *E. coli* JM109. The heat-stable extracts were prepared from each clone, and were used for the DNase assay. The DNA fragment from the positive clone was analyzed by DNA sequencing, which revealed the presence of 5 full-length ORFs in the 5-kbp fragment. These 5 ORFs were amplified by PCR directly from the genomic DNA of *P. furiosus*. Each amplified gene was cloned into the pGEM-T easy vector (Promega). The cloned genes were digested by either NdeI-BamHI or NcoI-BamHI, and were inserted into the corresponding sites of pET21a or pET21d. The constructed plasmids were designated as pPF2045, pPF2046, pPF2047, pPF2048, and pPF2049, respectively. *E. coli* cells were transformed with the plasmids, and heat-stable extracts were prepared from each clone and used for the DNase assay.

### Production and Purification of PfuExo I

*E. coli* BL21-codon plus (DE3)-RIL cells (Novagen) harboring pPF2046 were grown at 37°C, in 1 liter of LB medium containing 50 µg/ml ampicillin and 34 µg/ml chloramphenicol. When the *E. coli* culture reached an A_600_ of 0.52, expression of the gene encoding PfuExo I was induced by adding IPTG to 0.1 mM. After cultivation at 18°C for 24 hours, the cells were harvested by centrifugation, and the cell lysate was prepared by sonication in 40 ml of buffer A (50 mM Tris-HCl, pH 8.0, 10% (v/v) glycerol, 0.5 mM DTT, and 0.1 mM EDTA) containing 0.5 M NaCl and 1 mM PMSF. After centrifugation for 20 min at 23,700*×g*, the supernatant was incubated at 80°C for 30 min to remove most of the *E. coli* proteins, and then centrifuged again. To the heated supernatant, polyethyleneimine was added to 0.15% (w/v). The solution was stirred on ice for 30 min, and centrifuged for 10 min at 23,700*×g*. Ammonium sulfate was added to the supernatant to 80% saturation, and the solution was stirred on ice for 1 hour. The precipitate from the ammonium sulfate treatment was suspended in 8 ml of buffer A containing 1 M ammonium sulfate, and applied to a hydrophobic column (HiTrap phenyl, GE Healthcare UK Ltd), which was developed with a linear gradient of 1.0–0 M ammonium sulfate. The PfuExo I-containing fractions were applied to an anion-exchange column (HiTrapQ, GE Healthcare UK Ltd), after dialysis against buffer A containing 0.1 M NaCl. The column was developed with a 0.1–1.0 M NaCl gradient. The active fraction eluted at 0.15 M NaCl was diluted to 0.015 M NaCl, and applied to an affinity column (HiTrap heparin, GE Healthcare UK Ltd), which was developed with a linear gradient of 0.1–0.8 M NaCl. The eluted fractions were concentrated and stored at 4°C. The protein concentrations were determined by measuring the absorbance at 280 nm, with an extinction coefficient of 35,410 M^−1^ cm^−1^, which was obtained by the method described earlier [44].

### Exonuclease Assay

The heat-stable protein extracts or purified PfuExo I was incubated with 5 nM of various ^32^P-labeled substrate DNAs in a standard reaction mixture, containing 20 mM Tris-HCl (pH 8.8), 5 mM MgCl_2_, 50 mM NaCl, and 0.1 mg/ml BSA. Reaction time and temperature are described in each figure legend. The reactions were stopped with an equal volume of deionized formamide. These samples were subjected to polyacrylamide-8 M urea gel electrophoresis, and the degradation products were visualized by autoradiography.

### Endonuclease Activity

Purified PfuExo I (100 nM) was added to the reaction mixture containing 5 ng/µl of pBR322 or M13 mp18 ssDNA (Takara-bio), in the standard reaction condition for the exonuclease assay for 30 min at 65°C. The reactions were terminated by the addition of an equal volume of phenol. The reaction products were fractionated by electrophoresis on a 1% agarose gel, and the DNA was visualized by ethidium bromide staining.

### Western Blot Analysis

*P. furiosus* cells (4.5×10^11^ cells) were disrupted by sonication in 15 ml of the buffer containing 50 mM Tris-HCl, pH 7.0, 0.5 mM dithiothreitol, 0.1 mM EDTA, and 10% glycerol with proteinase inhibitor (Complete™, Roch Diagnostics GmbH), and the extract was obtained by centrifugation. The *P. furiosus* cell extract and the purified PfuExo I protein were separated by 14% SDS-PAGE, blotted onto PVDF membranes, and reacted with anti-PfuExo I antiserum, prepared by immunizing a rabbit with the recombinant PfuExo I protein. The bands were detected by using ECL (GE Healthcare UK Ltd), according to the supplier’s recommendations.

### Gel Filtration Analysis

The gel filtration analysis was performed using the SMART system (GE Healthcare UK Ltd). Purified recombinant PfuExo I (20 µM) was applied to a Superdex 200 3.2/30 column, pre-equilibrated with buffer (50 mM Tris-HCl, pH 8.0, 0.5 mM DTT, 0.1 mM EDTA, 10% glycerol, and 0.15 M NaCl). The molecular weight of PfuExo I was estimated from the elution profile of standard marker proteins, including thyroglobulin (670,000), γ-globulin (158,000), ovalbumin (44,000), and myoglobin (17,000).

### DNA Binding Activity of PfuExo I

Different concentrations (0–1,000 nM) of purified recombinant PfuExo I were incubated with 2.5 nM of various ^32^P-labeled substrate DNAs, in a reaction mixture containing 20 mM triethanolamine, 50 mM NaCl, 0.1 mg/ml BSA, and 0.1 mM EDTA, for 20 min at 37°C. The reaction mixtures were treated with 0.1% glutaraldehyde, to fix the protein and DNA. The protein-DNA complexes were fractionated by 4.5% native PAGE and visualized by autoradiography, using an FLA5000 bioimage analyzer (FUJIFILM).

### Computer Analysis of the Amino Acid Sequences

Search for the homologous sequences in the databases with BLAST was carried out at a website (http://blast.ncbi.nlm.nih.gov/Blast.cgi). CLUSTALW (http://www.genome.jp/tools/clustalw/) was used for the amino acid sequence comparison of the PfuExo I and other *Thermococcal* homologs. It should be noted that the genome sequence encoding the homolog in *P. horikoshii* lacked one nucleotide at 514^th^ from the start site of the ORF (PH0067, accession number; NP_142085.2), and this deletion caused a frame shift in the downstream of the ORF. Therefore, we used our own sequence data, which codes for the amino acid sequence with high homology in the entire ORF, as PH0067 for the comparison. The other sequences were picked up from the database as follows. PF2046; NP_5797751, PAB2293; NP_125767.1, TK1646; YP_184059.1.
